# Saudi national science, technology and innovation plan towards knowledge based economy

**DOI:** 10.1186/1471-2164-15-S2-O2

**Published:** 2014-04-02

**Authors:** Abdulaziz M Al-Swailem

**Affiliations:** 1 King Abdulaziz City for Science and Technology (KACST), Riyadh, Kingdom of Saudi Arabia

## 

National Science, Technology and Innovation strategic Plan (NSTIP) is among many other national plans followed in Saudi Arabia to shift its economy from oil based to knowledge based. The National Policy for Science and Technology, approved by the Council of Ministers in 1423 Hijri (2002 G), has defined 15 programs for localization and development of strategic technologies that are essential for the future development of the Kingdom of Saudi Arabia.

NSTIP is mandated to direct science research and technology development in the Kingdom of Saudi Arabia towards long-term development goals of the country. It is also responsible for strengthening ties between key components of the Science and Technology national plan, such as research and development centers, education and training organizations, companies, investors, innovators, technology suppliers, consulting firms and scientific media.

NSTIP also works closely with the education sector to ensure that science and technology education programs are in line with Science and Technology National Policy (STNP) requirements. They both also help in developing programs and institutes for higher studies in applied scientific and technical fields.

NSTIP promotes and develops financial support sources in addition to developing regulations that govern the performance of the policy. It also encourages and increases international cooperation in the development of the science and technology fields and it is responsible for the dissemination and distribution of scientific and technology information.

King Abdulaziz City for Science and Technology (KACST) monitors the implementation of NSTIP, plays a major role in executing the plan, chairs the steering committee, and publishes reports about the key performance indicators and many governing regulation.

